# Effectiveness of a community-based, multicomponent and case managed treatment for patients with severe schizophrenia

**DOI:** 10.1192/j.eurpsy.2025.709

**Published:** 2025-08-26

**Authors:** J. J. Fernández-Miranda, S. Díaz-Fernández, F. López-Muñoz

**Affiliations:** 1 AGCSM V- Hospital de Cabueñes, SESPA, Gijón; 2 Health Sciencies, Universidad Camilo J. Cela, Madrid, Spain

## Abstract

**Introduction:**

Case management is a model of community intervention in people with severe mental illness.

**Objectives:**

To explore the treatment adherence and effectiveness of patients with severe schizophrenia undergoing treatment in a community-based, case management program (CMP) with an integrated pharmacological and psychosocial approach compared to the standard treatment.

**Methods:**

An observational, longitudinal study was conducted with a ten-year follow-up of patients with severe schizophrenia (CGI-S ≥ 5) treated in mental health units (MHUs) or on a CMP (N = 688). All causes of treatment discontinuation, psychiatric hospital admissions, suicide attempts, and antipsychotic (AP) medications were recorded. Clinical severity was assessed with the CGI-S.

**Results:**

Adherence to the CMP was higher than to the standard treatment (p < 0.001). There were fewer hospital admissions and suicide attempts on the CMP than in standard care (p < 0.001). Clinical severity decreased more in the CMP than in MHUs (p < 0.005). Long-acting injectable (LAI) AP medication was more closely related to these outcomes than oral APs (p < 0.001) in both settings, but especially on the CMP.

**Treatment clinical outcomes after 10-year follow-up**

**Table:** 

***N = 688***	**Mental Health Units (N = 344)**	**Case Management Program (N = 344)**	**χ** ^**2**^ **; P value**
**Treatment discontinuation (N (%))**	150 (43.6)	42 (12.1)	26.16; < 0.0001
**CGI-S (Av (SD))**	3.9 (1.1)	3.1 (0.9)	7.63; < 0.005
**Hospitalization (N (% ))**	160 (46.5)	60 (17.4)	10.54; < 0.0001
**Hospitalization (Av (SD))**	3.2 (3.4)	0.9 (0.3)	13.23; < 0.0001
**Involuntary hospital. (N (% ))**	34 (9.9)	5 (1.4)	28.01; < 0.0001
**Involuntary hospital. (Av (SD))**	0.5 (0.3)	0.01 (0.2)	21.31; < 0.0001
**Suicide attempt (N (%))**	85 (24.7)	20 (5.8)	10.54; < 0.0001
**Num. suicide attempts (Av (SD))**	0.3 (0.1)	0.07 (0.02)	11.32; < 0.0001

*N: number of patients %: percentage of patients Av: average SD: standard deviation*

*: basal (at beginning of program) **: standard treatment ***: Program treatment

**Conclusions:**

The treatment of patients with severe schizophrenia in a multicomponent, case-managed program recorded higher compliance and effectiveness compared to standard care. Treatment with LAI antipsychotics was linked to these outcomes. A combination of case management, psychosocial approach, and LAI AP medication contributed more to the achievement of clinical goals in these patients than the standard treatment and oral APs.

**Disclosure of Interest:**

None Declared

